# Clinical Performance of Computer-Aided Design (CAD)/Computer-Aided Manufacturing (CAM) Resin Nanoceramic Endocrowns in Restoring Molars: An In Vivo Study

**DOI:** 10.7759/cureus.64252

**Published:** 2024-07-10

**Authors:** Sewar Ibrahem, Luai Morad, Hassan A Husein

**Affiliations:** 1 Fixed Prosthodontics, Faculty of Dental Medicine, Damascus University, Damascus, SYR

**Keywords:** clinical performence, color stability, cad/cam, nanoceramic, endocrown

## Abstract

Background

It is difficult to determine the optimal method for restoring endodontically treated teeth, as several factors affect this decision. Functional requirements and the amount of remaining coronal tissue are considered the most important factors in choosing the best restoration for those teeth. Endocrown was introduced as a conservative alternative for endodontically treated and coronally damaged teeth.

Aim

The aim of this study is to assess the clinical performance of the nanoceramic system for molar endocrowns by evaluating color change, gingival condition, prosthesis integrity, and the presence of secondary caries.

Materials and methods

The sample consisted of 20 endocrowns. The teeth were prepared with at least 2 mm of wall thickness and a cavity depth of 4 mm from the occlusal surface. The final impression was taken, and the prostheses were adhered using dual-cure resin cement. It was clinically evaluated according to clinical success criteria (United States Public Health Service) in terms of color stability, gingival indexes, integrity of the restoration, and the presence of secondary caries after a week, three months, six months, and a year. The statistical study was conducted using IBM SPSS Statistics for Windows, Version 23.0 (Released 2015; IBM Corp., Armonk, NY, USA), and the results were considered statistically significant at the 95% level of significance. The Friedman test was used to study the significance of the differences in the average values of color change, plaque index, and the integrity of the prostheses during follow-up periods.

Results

The study showed a gradual increase in the degree of color change with follow-up periods. Furthermore, there was no significant difference in the incidence of gingival changes, the integrity of the prosthetic margins, or the occurrence of secondary caries during the follow-up periods.

Conclusions

Within the limitations of this study, an endocrown made of CeraSmart nanoceramic is an acceptable option for crowning decayed and endodontic-treated molars with acceptable clinical performance.

## Introduction

Indirect partial restorations, such as inlays, onlays, overlays, endocrowns, and full crowns, help preserve and strengthen the remaining dental tissues damaged as a result of decay or fracture.

In 1995, Pissis presented the idea of endocrown (integrated into the pulp chamber) based on merging the structure with the overlying layer as a single-mass prosthesis, covering the occlusal surfaces and extending into the pulp chamber, and relying on the axial walls as mechanical retention and on adhesive cement as microscopic mechanical retention [1]. Endocrown is characterized by ease of preparation, high fracture resistance, lack of laboratory and clinical procedures, and low cost, in addition to cosmetic properties [2].

Extensive development in nanotechnology in the fields of materials science and engineering has occurred over the past decade. However, such developments are not surprising, as it has become possible to adapt these nanostructured materials and integrate them into biomedical devices and biological systems, as most biological systems, including viruses, membranes, and protein complexes, naturally appear as nanostructures. Nanostructured materials refer to certain materials that have the structure or size of composite structures in the range of 1-100 nanometers [3].

The term nanoceramics is used to describe ceramic materials consisting of at least one component with dimensions in the nanorange (1-100 nm), which were discovered in the 1980s and have received great interest from researchers. Nanoceramics are used in many current technologies due to their excellent biocompatibility, strength, abrasion resistance, and small size [4]. Nanoceramics have physical, chemical, and mechanical properties that are different from those of other materials, such as traditional ceramics, plastics, and metals. The type, size, and quantity of the raw material from which nanoceramic is made often determine the level of distinctive and improved properties, which include surface properties, mechanical properties, and processing, including machinability, superplasticity, strength, toughness, and bioactivity. Nanoceramics are extremely strong and show great resistance to bending and compression.

Two categories of materials have been used to produce computer-aided design (CAD)/computer-aided manufacturing (CAM) prosthetics: traditional porcelain and indirect composite materials. While porcelain has generally superior mechanical and aesthetic properties, resin composite materials may offer multiple advantages regarding their manufacturability and intraoral reparability [5].

The category of composite materials manufactured from CAD/CAM blocks is divided into two subcategories according to their microstructure: the first contains dispersed fillers, while the second contains a structure containing polymer-infiltrated ceramic network (PICN) [6,7].

CeraSmart (CS) is known as a PICN hybrid ceramic composed of a flexible ceramic matrix with an even distribution of nanoceramics. It is a resinous material composed of silica and barium glass nanoparticles (71% by weight) and a polymer content of Bis-MEPP, UDMA, and DMA (29% by weight), with a flexural strength equivalent to 238 MPa [6,7].

Hence, this study aimed to assess the clinical performance of CS endocrown manufactured using the CAD/CAM technique.

## Materials and methods

Patient recruitment

The sample size was determined using G*Power software, and patients with destruction of the first or second lower molars were included. The sample comprised 20 crowns for patients attending the Department of Fixed Prosthodontics at the Faculty of Dental Medicine, Damascus University, Syria, who met the study’s inclusion criteria. Endocrowns were fabricated from CS (nanoceramic material) using the CAD/CAM technique, adhered to with the same adhesive, and clinically evaluated based on the United States Public Health Service (USPHS) clinical success criteria.

The inclusion and exclusion criteria are presented in Table 1.

**Table 1:** Inclusion and exclusion criteria

Inclusion criteria	Exclusion criteria
Patient with good oral hygiene and a low caries rate	Premolars and third molars
The periodontal tissues are healthy	
Having a corresponding tooth	Truisms and temporomandibular joint problems
Underwent endodontic treatment	
Absence of abnormal functional habits such as teeth grinding	Low thickness of the remaining tissues
Possibility of follow-up for at least one year	

Following detailed explanations of all stages of the prosthetic work and procedures during follow-up periods for each patient, a special form was completed. This form encompassed general examinations, dental assessments, treatment plans, and follow-up details. Informed consent was obtained or waived by all participants in this study, and approval was granted by the Local Research Ethics Committee of the Faculty of Dentistry, Damascus University (approval number UDDS-1550-16022021).

Teeth preparation

**Figure 1:**
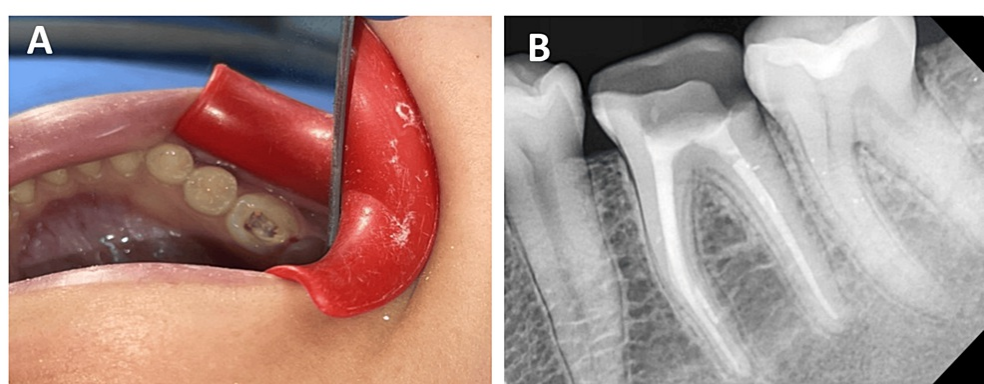
Evaluation of the tooth after endodontic treatment: (A) Clinical examination. (B) Radiographic examination

After endodontic treatment (Figure 1), the teeth were prepared according to specified criteria by reducing the occlusal surface with a diamond wheel, leaving the remaining portion 2 mm above the cementoenamel junction.

**Figure 2:**
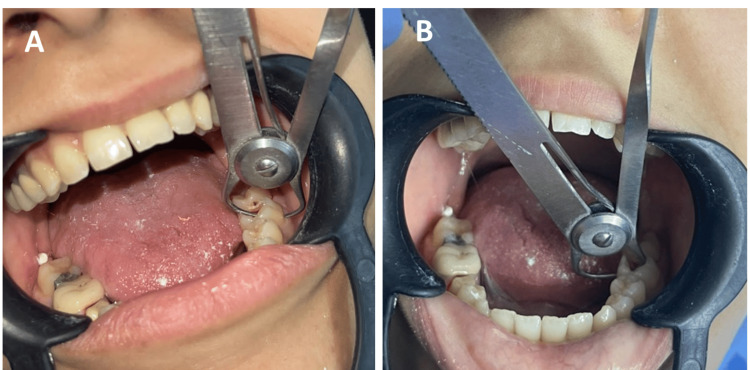
Measurement of the remaining wall thickness: (A) Buccal wall. (B) Lingual wall

The internal axial walls were prepared using an 8-degree diamond cone bur, ensuring no interference with the bottom of the pulp chamber. The remaining walls were maintained at a thickness of at least 2 mm, and the cavity depth was prepared to be 4 mm from the occlusal surface (Figure 2).

**Figure 3:**
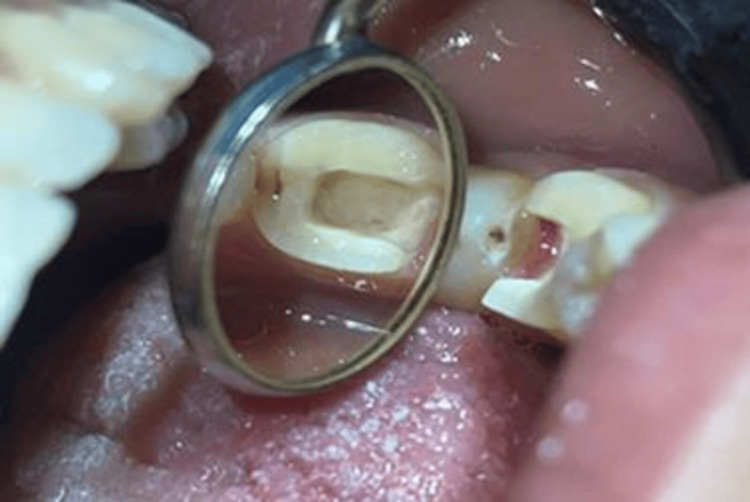
Sealing of the canal orifice

The guttapercha cones were removed from the canal orifices to a depth not exceeding 2 mm, and the orifices were filled with a resin sealer along with the cavity areas (Figure 3). Subsequently, a layer of glass ionomer cement was applied to create a flat base at the bottom of the chamber.

Final impression

**Figure 4:**
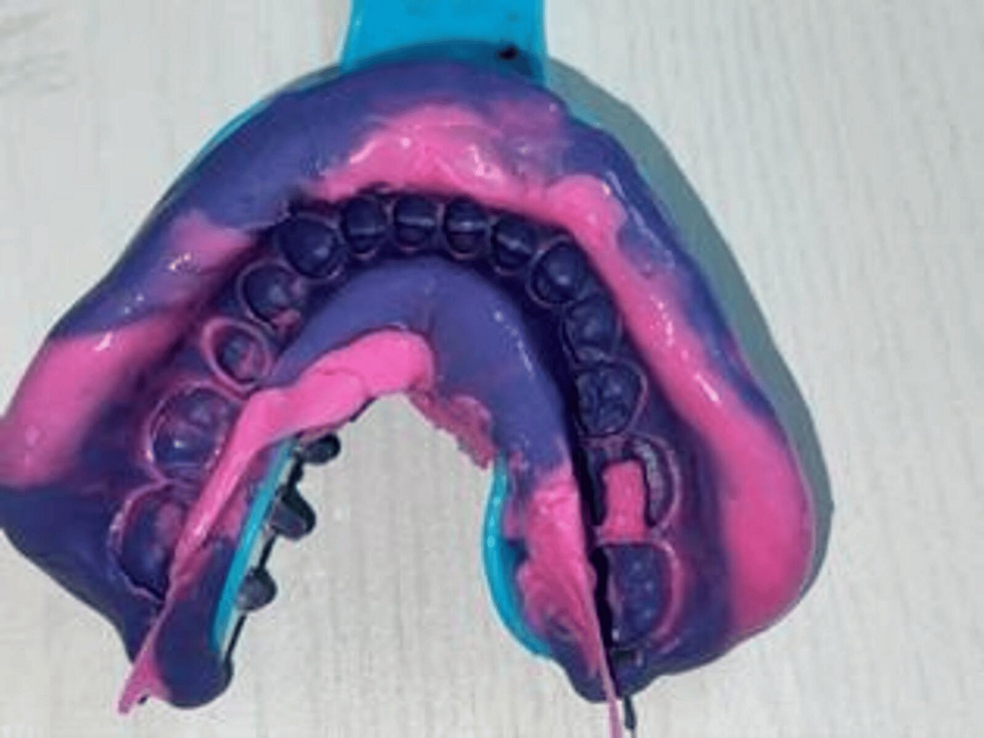
Final impression

The final impression of the entire dental arch was obtained using a two-stage putty-wash impression technique with additional silicone and perforated metal trays (Figure 4). The jaw relationship was recorded in the maximum intercuspal position using a wax basal plate.

An appropriate amount of temporary composite (Tokuyama Corporation, Tokyo, Japan) was applied and condensed onto the cavity floor using a metal condenser to ensure complete coverage of the edges. It was then light-cured for 20 seconds on the occlusal surface.

Try-in and final cementation

The temporary prosthesis was removed, and the teeth were cleaned of any residual composite. A clinical try-in was then performed to verify internal adaptation using light-cured silicone, ensuring a thin and uniform layer, and marginal adaptation was checked with a dental probe. Additionally, the form and color of the prostheses were assessed visually, involving the patient’s opinion. Finally, contact points were identified using dental floss.

**Figure 5:**
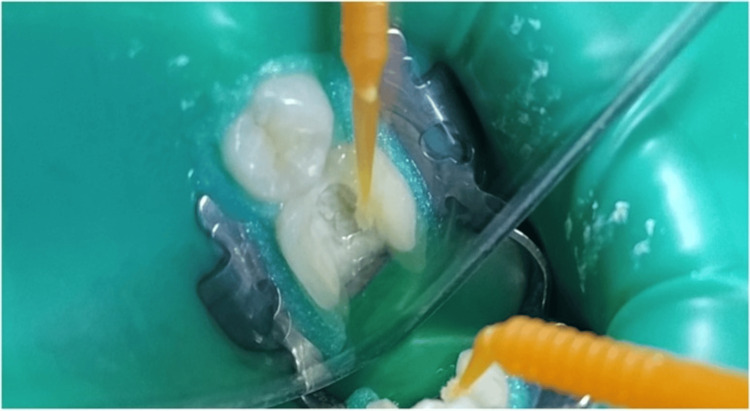
Application of the bonding agent

The necessary modifications were made chairside and communicated to the dental lab for specific adjustments. Subsequently, the endocrowns were adhered to using the approved adhesive system, following these steps:

First, the surfaces of the teeth prepared for endocrowns were treated as follows: a mouth opener was fitted, dental cavities were cleaned with fluoride-free pumice powder, and they were washed with a stream of water and air. Non-impregnated gingival sutures of size 000 were applied to control gingival fluids, followed by fitting a rubber dam. Adjacent teeth were isolated with thin strips of Teflon to protect them from adhesive procedures and facilitate the cleaning of excess adhesive material. Next, the tooth enamel surfaces were etched with 37% phosphoric acid for 30 seconds, thoroughly washed with a stream of water, and dried with a gentle air stream. Finally, bonding material was applied to the prepared tooth surface using a single-use bond brush and then light-cured (Figure 5).

**Figure 6:**
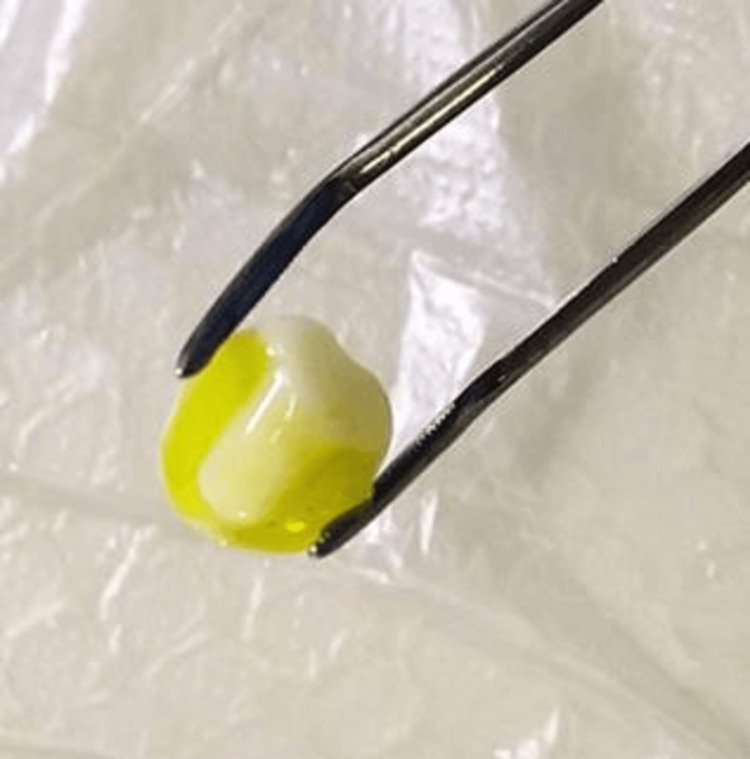
Etching of the inner surface of the prostheses with hydrofluoric acid

Second, the inner surface of the endocrown was cleaned, washed with 90% alcohol, followed by water, and dried using an air stream. Subsequently, it was etched with 5% hydrofluoric acid for 60 seconds (Figure 6), rinsed with water for 20 seconds, and dried with an oil-free air stream. Finally, silane was applied for 60 seconds and dried with an air stream.

**Figure 7:**
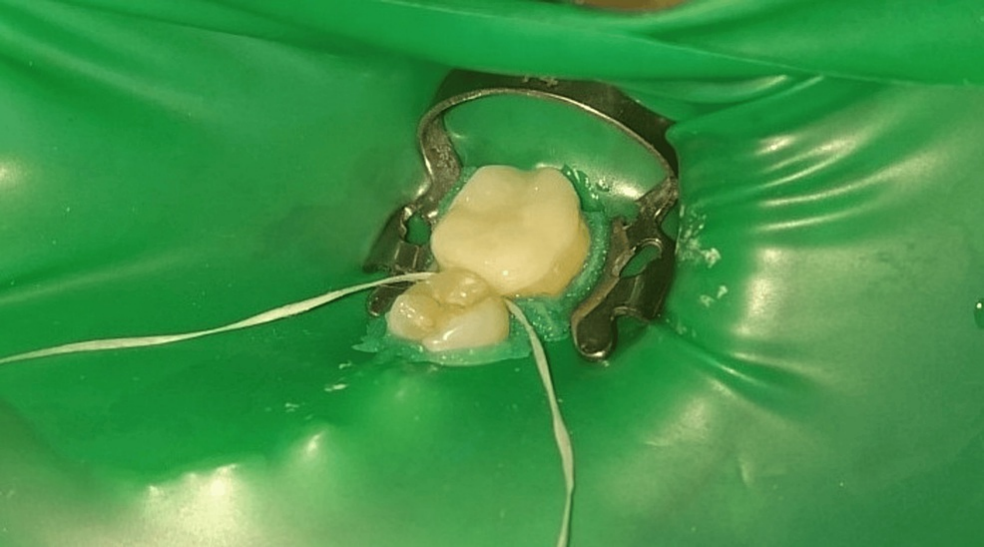
Final cementation and removal of excess cement

Third, the application of the resin cement (Multilink N, Ivoclar Vivadent Inc., Schaan, Liechtenstein) proceeded as follows: the base and accelerator materials of the dual-cure resin cement were mixed on special paper provided by the company. Using a spatula, the mixture was transferred to the surface of the endocrown. The endocrown was then placed on the prepared tooth with light pressure until it was properly seated. Next, it was light-cured for two seconds, excess cement was removed with a blunt tool, and final curing was completed for 60 seconds (Figure 7).

**Figure 8:**
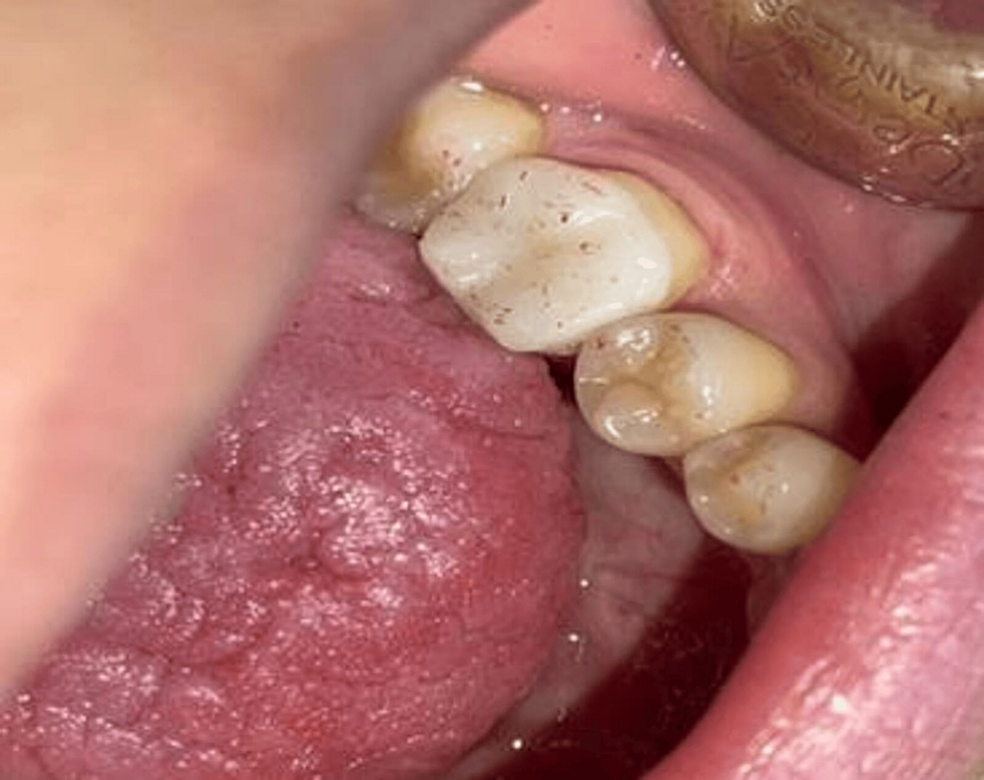
The final result after completion of the procedure

Following this, an oxygen barrier layer was applied to the crown edges. The edges of the crown were refined using ultra-fine diamond burs and fine grinding strips for the adjacent areas. Occlusal adjustments were then made using 8-micron articulating paper and fine diamond burs (Figure 8).

Finally, the modified points were finished and polished using special porcelain intraoral rubber heads. Data for each case were recorded after the final cementation stage on a designated research form. The endocrowns were evaluated before cementation, immediately after final cementation, and at intervals of one week, one month, three months, six months, and one year thereafter.

Gingival inflammation

The gingival inflammation index used in this study is as follows: 0 = normal gingiva; 1 = mild inflammation characterized by slight changes in color and slight edema, with no bleeding on probing; 2 = moderate inflammation presenting with redness, edema, and glazing, or bleeding on probing; and 3 = severe inflammation marked by pronounced redness and edema, with a tendency toward spontaneous bleeding and possible ulceration [8].

Color changing

The color change was measured using an electronic measuring method (VITA Easyshade compact, Vident). The evaluation criteria were as follows: Alpha indicated a color change of less than 1 (Delta E<1), Bravo indicated a color change ranging between 1 and 3.7 (3.7<Delta E>1), and Charlie indicated a color change of more than 3.7 (Delta E > 3.7).

Prostheses integrity

Marginal integrity was assessed using specific criteria: Alpha 1 denoted no noticeable cracks or slits, ensuring the probe did not catch in any areas like slits, extensions, or missing edges. Alpha 2 indicated the probe catching as it moved from the tooth to the restoration. Alpha 3 indicated catching as it moved from the restoration to the tooth. Bravo 1 described the formation of cracks or fissures during probing that extended to less than 50% of the restoration edges, whereas Bravo 2 extended to more than 50%. Charlie represented cracks or fissures exposing the underlying dentin or base, and Delta indicated significant issues such as movement, breakage, or missing parts of the restoration.

Secondary caries

The assessment for crown margins was conducted as follows: Alpha was recorded in the absence of caries, while Bravo was recorded if caries were present.

Statistical analysis

The data were entered into a computer and analyzed using IBM SPSS Statistics for Windows, Version 23.0 (Released 2015; IBM Corp., Armonk, NY, USA). Statistical analyses considered p-values significant at the 95% confidence level. The p-value results were adopted at the significance level of 95%.

The Friedman test was employed to assess the significance of differences in average color change values, incidence of gingival inflammation, and prosthesis integrity across the follow-up periods.

## Results

Sample description

The study sample comprised 20 endocrowns, with patients aged between 35 and 50 years (five males and three females). The average age of male patients was 45.69 years, while female patients had an average age of 43.57 years (Table 2).

**Table 2:** Arithmetic mean and standard deviation of patients’ ages (in years) according to patient sex

Patient gender	Number of patients	Arithmetic mean (years)	Standard deviation
Male	13	45.69	1.49
Female	7	43.57	0.95
Total	20	44.63	1.22

Color change

**Figure 9:**
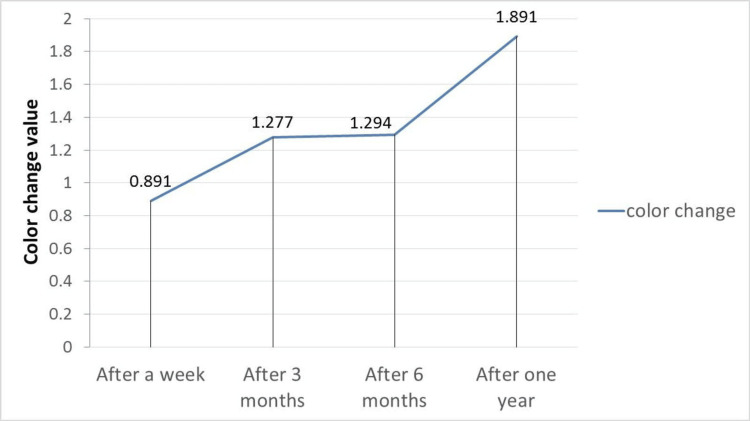
Color change in the study sample

Table 3 shows the descriptive statistics for the color change values, including the arithmetic mean, standard deviation, and the highest and lowest values for the study sample according to the follow-up period. The averages showed a gradual increase in color change values with an increasing follow-up period (Figure 9).

**Table 3:** Descriptive statistics of color change values in the study sample according to the follow-up periods

Follow-up periods	Arithmetic mean	Standard deviation	Maximum	Minimum
After a week	0.891	0.089	1.06	0.76
After three months	1.277	0.41	1.34	1.21
After six months	1.294	0.342	1.37	1.24
After one year	1.891	0.432	1.97	1.81

The Friedman test was employed to assess the significance of differences in the average color change values of the endocrowns across follow-up periods. The results indicated a p-value lower than 0.05 at a 95% confidence level, indicating statistically significant differences in color change values between follow-up periods. Subsequently, the Wilcoxon test was used to analyze the significance of differences in color change values between each pair of time points studied (Table 4).

**Table 4:** Results of the Wilcoxon test for differences in average color change values between each of the two follow-up periods

Variable	Follow-up		z-value	p-value	Significance
Color change	One week	Three months	-3.922	0	Significant differences found
		Six months	-3.922	0	Significant differences found
		One year	-3.922	0	Significant differences found
	Three months	Six months	-1.199	0.231	No significant differences
		One year	-3.922	0	Significant differences found
	Six months	One year	-3.926	0	Significant differences found

Table 4 illustrates significant differences in color change values across all follow-up periods except between the three-month and six-month periods. In terms of arithmetic averages, the color change values showed a gradual increase, starting at 0.891 after one week and rising to 1.891 after one year.

Gingival state

**Figure 10:**
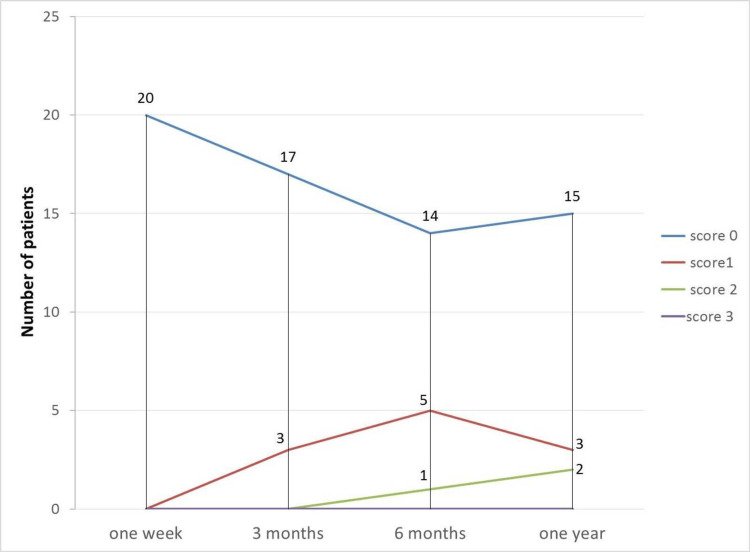
Plaque index during the follow-up periods

A descriptive analysis was conducted on bacterial plaque formation scores during the follow-up periods. Initially, all samples showed a score of 0 for bacterial plaque index after one week. By three months, three patients had their first instance of bacterial plaque, whereas 17 patients maintained a score of 0. At six months, the number of patients with a score of 0 decreased to 14, while five patients had a score of 1, and one patient had a score of 2. After one year, the number of patients with a score of 2 increased to 2, three patients had a score of 1, and 15 patients had no bacterial plaque. No patients exhibited a score of 3 for bacterial plaque formation (Table 5, Figure 10).

**Table 5:** Descriptive statistics of the bacterial plaque index according to the follow-up period

Follow-up periods	Number of patients	Plaque index				Percentage			
		Score 0	Score 1	Score 2	Score 3	Score 0	Score 1	Score 2	Score 3
A week	20	20	0	0	0	100%	0%	0%	0%
Three months	20	17	3	0	0	85%	15%	0%	0%
Six months	20	14	5	1	0	70%	25%	5%	0%
One year	20	15	3	2	0	75%	15%	10%	0%

The Friedman test was utilized to examine the effect of time on plaque accumulation in the sample. The significance level (P) was found to be 0.109, which is higher than 0.05. This indicates that, at a 95% confidence level, there are no statistically significant differences observed when comparing plaque accumulation between the follow-up periods.

Marginal integrity

Descriptive statistics were conducted to assess the integrity of the margins across all follow-up periods. It was observed that all samples maintained intact margins after a week, three months, six months, and one year, except for two cases of restoration fracture (Table 6).

**Table 6:** Descriptive statistics for the edge integrity standard in the study sample according to the observation period

Follow-up periods	Number of patients	Marginal integrity						
		Alpha 1	Alpha 2	Alpha 3	Bravo 1	Bravo 2	Charlie	Delta
A week	20	20	0	0	0	0	0	0
Three months	20	18	0	2	0	0	0	0
Six months	20	20	0	0	0	0	0	0
One year	20	20	0	0	0	0	0	0

The Friedman test was employed to examine the effect of time on marginal integrity. The significance level (P) was calculated as 0.109, which is higher than 0.05, indicating that at the 95% confidence level, there were no statistically significant differences observed in marginal integrity over time.

Secondary caries

Table 7 presents descriptive statistics for the secondary caries criterion across all follow-up periods. It was observed that the dental caries criterion was consistently categorized as Alpha throughout all periods, indicating that all teeth were free of caries after one week, three months, six months, and one year.

**Table 7:** Descriptive statistics for the secondary caries criterion in the study sample according to the follow-up period

Follow-up periods	Number of patients	Secondary caries criterion		Percentage	
		Alpha	Bravo	Alpha	Bravo
A week	20	20	0	100%	0%
Three months	20	20	0	100%	0%
Six months	20	20	0	100%	0%
One year	20	20	0	100%	0%

## Discussion

Many studies have recommended the use of the endocrown as a clinically viable prosthetic option for restoring lost crown structures in endodontically treated posterior teeth. Laboratory studies have highlighted its resistance to compressive forces and the simplicity of its application procedures [9-11]. Therefore, this research was initiated to gather more precise information about this prosthetic option using new materials.

The upper molars were excluded from the sample based on findings from previous studies indicating a difference in marginal fit compared to the more precise lower molars. To maintain accuracy, standards, and uniformity, only lower molars were included, with a focus on ensuring adjacent and opposing teeth were present and intact or crowned. Third molars were excluded due to accessibility challenges and concerns about procedure accuracy, similar to the rest of the sample, which involved preparation, impression, and adhesiveness. Premolars were also omitted due to documented variations in endocrown performance on premolars, attributed to the small surface area of the pulp chamber affecting stability [12]. Additionally, the anatomical shape of premolars, characterized by a higher crown height-to-width ratio, creates a lever arm effect that can potentially compromise bonding and lead to prosthesis loosening [13]. Therefore, lower first or second molars were selected to minimize variables in the study.

Hybrid nanoceramic was chosen for manufacturing modified crowns in this research due to its flexible properties compared to traditional porcelain types, its high aesthetic appeal, and its robust mechanical properties, which make it well-suited for such applications. Clinically, it offers a wear coefficient similar to that of natural teeth, owing to its polymer matrix, which occupies approximately 30%. Additionally, its single-layer anatomical restoration design eliminates the need for covering layers seen in zirconia and some other porcelain restorations, addressing potential weaknesses associated with covering porcelain such as spalling and reduced fracture resistance [14].

Dual-curing resin cement was selected because it is recommended for cases where the occlusal thickness of the prostheses prevents sufficient light penetration for complete curing with light-curing systems. This cement ensures reliable polymerization throughout the restoration, addressing the challenges posed by inadequate light-intensity penetration [15].

Color changes are represented by ΔE, defined as the numerical difference between the L*a*b* coordinates of two colors. The detectability thresholds were established: ΔE < 1 is undetectable to the human eye; 1.0< ΔE < 3.3 is only seen by a skilled person and considered clinically acceptable; and ΔE > 3.3 is considered easily observable and clinically unacceptable [16].

Many studies have explored the impact of colorants on the color stability of various hybrid ceramics, offering insights into their clinical performance and maintenance of aesthetic properties. They indicate that prosthetics made of nano-hybrid ceramics exhibit slightly higher color change compared to other hybrid and traditional ceramics, yet all values typically fall within the clinically acceptable range (1.0 < ΔE < 3.3) [16-18]. Similarly, the findings of the present study show that the color change values of the prostheses remained within this clinically acceptable range, with the highest recorded value at 1.97.

In contrast, Arif suggested avoiding nanoceramic materials for veneers in aesthetically demanding areas, particularly for individuals who consume coffee frequently, due to the significant color changes that can occur [19].

Color changes can result from various factors, such as mechanical or chemical abrasion, as well as exposure to saliva, foods, and beverages [20]. The material’s ability to withstand occlusal wear is crucial for long-term success. Advances in filling systems, including improvements in the mechanical and physical properties of resin matrix materials, have been significant [21]. Smaller filler particle sizes contribute to better wear resistance, whereas larger particles can increase surface prominence and susceptibility to scratching, thereby potentially causing color changes [22]. Furthermore, manufacturing procedures for hybrid ceramics, whether resin-based or not, play a significant role in color stability. The structure and properties of ceramic materials can be influenced by factors such as processing conditions, temperature, and pressure during the manufacturing process [23].

No cases of secondary caries were observed during the follow-up period. This outcome could be attributed to the relatively short duration of follow-up as well as the ease of cleaning facilitated by all endocrown margins being positioned above the gingival level. Additionally, all patients maintained good oral hygiene practices.

This finding aligns with Kaya and Alp’s study, where they evaluated the clinical performance of three endocrowns in a single patient for one-year post-cementation. They reported no observations of prosthetic or tooth fractures, secondary caries, or debonding of the prostheses [24]. In contrast, Vervack et al. reported the extraction of a tooth fitted with a nanoceramic endocrown due to secondary caries after a five-year follow-up period [25].

No cases of partial or complete prosthesis fracture were observed in any of the samples during the follow-up period. This can be attributed to the manufacturing method of all endocrowns using the CAD/CAM single-layer technique. Crowns made from flexible hybrid ceramic do not require additional layers of porcelain or resin. Moreover, their low elasticity factor, similar to that of dental tissue, allows them to absorb applied forces without concentrating or transferring them to specific points.

The results of this study align with those reported by El-Ma'aita et al. in 2022 regarding the durability of hybrid ceramic endocrowns. They found a durability rate of 89.5% after two years of follow-up, with two cases experiencing failure (one due to restoration fragmentation and the other due to restoration fracture) out of 20 cases. Additionally, the study indicated a 100% patient satisfaction rate at the end of the study [26].

Salem et al. conducted a study comparing the clinical performance of endocrowns made of lithium disilicate or nano-hybrid ceramic. Over a three-year follow-up period, the USPHS clinical success rate for hybrid porcelain crowns was initially 100% Alpha in the first two years but decreased to 80% Alpha and 20% Bravo in the third year. The evaluation assessed marginal adaptation, color change, fracture, and restoration stability. It is noteworthy that the hybrid porcelain used, Grandio, differs in composition from the CS material, containing a high percentage of fillers (86% by weight) [27].

In this study, consistent gingival health outcomes were observed for the endocrowns. The success can be attributed to meticulous preparation techniques that avoided proximity to the gingival tissues, careful selection of patients with good oral hygiene habits, and thorough finishing and polishing of the prosthetic gingival margins. These measures ensured well-fitted endocrowns with smooth margins, facilitating easy maintenance and cleaning for the patients.

The present study has its limitations, including a relatively short follow-up period and a modest sample size. Consequently, additional long-term studies are warranted to further validate the findings of this research.

## Conclusions

Given the study’s limitations, endocrowns crafted from nanoceramic using CAD/CAM technology emerge as a viable choice for restoring decayed and endodontically treated molars. This option requires careful patient selection and precise adhesive techniques. The crowns demonstrated clinical reliability in terms of prosthesis integrity, color stability, and plaque accumulation.
